# Floral Sonication is an Innate Behaviour in Bumblebees that can be Fine-Tuned with Experience in Manipulating Flowers

**DOI:** 10.1007/s10905-016-9553-5

**Published:** 2016-04-15

**Authors:** Tan Morgan, Penelope Whitehorn, Gillian C. Lye, Mario Vallejo-Marín

**Affiliations:** Biological and Environmental Sciences, University of Stirling, Scotland, FK9 4LA UK; Natural Power Consultants, Ochil House, Springkerse Business Park, Stirling, Scotland FK7 7XE UK

**Keywords:** *Bombus terrestris*, bumblebee, buzz pollination, learning, pollen foraging, sonication

## Abstract

Bumblebees demonstrate an extensive capacity for learning complex motor skills to maximise exploitation of floral rewards. This ability is well studied in nectar collection but its role in pollen foraging is less well understood. Floral sonication is used by bees to extract pollen from some plant species with anthers which must be vibrated (buzzed) to release pollen. Pollen removal is determined by sonication characteristics including frequency and amplitude, and thus the ability to optimise sonication should allow bees to maximise the pollen collection. We investigated the ability of the buff-tailed bumblebee (*Bombus terrestris*) to modify the frequency and amplitude of their buzzes with increasing experience manipulating flowers of the buzz-pollinated plant *Solanum rostratum*. We analysed flight and feeding vibrations generated by naïve workers across feeding bouts. Feeding buzzes were of a higher frequency and a lower amplitude than flight buzzes. Both flight and feeding buzzes had reduced amplitudes with increasing number of foraging trips. However, the frequency of their feeding buzzes was reduced significantly more than their flight buzzes as bumblebee workers gained experience manipulating flowers. These results suggest that bumblebees are able to modify the characteristics of their buzzes with experience manipulating buzz-pollinated flowers. We discuss our findings in the context of bumblebee learning, and the current understanding of the optimal sonication characteristics for releasing pollen in buzz-pollinated species. Our results present a tantalising insight into the potential role of learning in floral sonication, paving the way for future research in this area.

## Introduction

Bumblebees (*Bombus* spp.) play an important role in providing pollinator services in both natural and agricultural systems (Goulson 2003; Goulson et al. 2008). The genus *Bombus* (Apidae) comprises approximately 250 species of medium-sized to large bees, distributed around the world (Michener 2007). These species rely on pollen and nectar as food sources, collecting these floral rewards from a diverse range of plant species (Sladen 1912; Goulson 2010). Previous studies have shown that bumblebees are able to learn to manipulate flowers with different morphologies (Heinrich 1979; Laverty 1980; Laverty and Plowright 1988; Laverty 1994; Raine and Chittka 2007, 2008) and across different contexts (Biernaskie et al. 2009), and that they can even learn from other individuals of the same (Leadbeater and Chittka 2008) or different species (Goulson et al. 2013). The ability to adjust their behavioural repertoire to manipulate flowers and collect resources from diverse plant species allows bumblebees to maximise the exploitation of floral resources over time, across different environments, and in distinct plant communities.

Bumblebees can learn in order to exploit floral resources more proficiently, and at a faster rate, with increasing experience in handling flowers (Heinrich 1979; Laverty 1980; Laverty and Plowright 1988; Laverty 1994; Raine and Chittka 2007, 2008). Previous work has shown that the amount of experience required to achieve maximum proficiency increases with increasing floral complexity (Laverty 1980, 1994). To date, most research on bumblebee learning has focused on behaviours displayed with relatively simple natural and artificial flowers in which nectar is used as the main reward (but see Raine and Chittka 2007; Kitaoka and Nieh 2009; Konzmann and Lunau 2014; Lunau et al. 2015; Muth et al. 2016). In contrast, less is known about whether and how bumblebees learn to manipulate complex flowers in which pollen is the main or only reward. The optimal method for pollen removal varies among plant species as a result of differing floral structure, anther morphology, and pollen properties (Buchmann 1983; Thorp 2000).

In most flowering plants, anthers split lengthwise to passively release pollen, which is then available for collection by pollinators (Buchmann 1983). However, in a sizeable number (around 20,000 species across a diverse suite of plant taxa), pollen is concealed within poricidal anthers (Buchmann 1983). These non-dehiscent, conical or tubular anthers have an apical pore through which pollen is expelled only when the anthers are vibrated (Buchmann 1983; Harder and Barclay 1994; De Luca and Vallejo-Marín 2013). Some floral visitors, including *Bombus*, can release pollen from these structures using a process known as sonication (Buchmann 1983). During sonication, the bee wraps its body around the anthers, while often holding them with its mandibles, and vibrates its thoracic muscles (the same muscles used in flight) at high frequencies. The vibrations are transmitted from the bee’s body to the anther, thereby releasing pollen.

The process of sonication requires the integration of a complex set of behaviours that involve the production of vibrations of high frequency. Previous studies have provided some evidence that the ability to produce sonication buzzes in the context of pollen extraction is innate in bumblebees (King 1993), and that learning plays an important role in allowing bumblebees to handle buzz-pollinated species effectively (Laverty 1980). However, whether and to what extent bumblebees can modify the characteristics of their buzzes, particularly the frequency and amplitude, in response to their experience in visiting flowers with poricidal anthers is unknown. Sonication buzzes generated during feeding differ in acoustic properties to vibrations produced under other contexts, such as defence buzzes (De Luca et al. 2014) and vibrations produced during flight (King et al. 1996). This raises the possibility that the characteristics of sonication buzzes can, to some extent, be actively modified by bees. The solitary bee species *Xylocopa frontalis* buzzes at slightly different frequencies when removing pollen from two different buzz-pollinated *Solanum* species, suggesting that such adjustments may be possible (Burkart et al. 2011). The ability to adjust the type of vibrations produced during pollen collection could allow bees to maximise the amount of pollen collected, e.g., by increasing the number of pollen grains released per buzz (Harder and Barclay 1994; De Luca et al. 2013).

Here we investigated whether bumblebees (*Bombus terrestris*) modify the characteristics of their sonication buzzes as they gain experience in sonicating on flowers. We used naïve bees that had never visited a flower and exposed them to flowers of *Solanum rostratum* (Solanaceae), a buzz-pollinated species with poricidal anthers. We recorded sonication buzzes during floral visitation and flight in consecutive visits to flowers over multiple days. By analysing the behaviour of these naïve bees, and the acoustic properties (frequency and amplitude) of their flight and feeding buzzes, we addressed two specific questions: (1) Is the ability to sonicate to elicit pollen release innate? (2) Do bees modify the characteristics of their feeding buzzes (frequency and amplitude) as they gain experience in visiting flowers with poricidal anthers?

## Methods

Our experiment required bees which had no previous experience of visiting flowers. We therefore used a colony of captive-bred British buff-tailed bumblebees, *Bombus terrestris audax.* The colony consisted of approximately 50 naïve workers at the start of the experiment (Biobest, Westerlo, Belgium). The colony was maintained under laboratory conditions (22–25°C) with natural light supplemented by both fluorescent and incandescent lighting. For feeding, the colony was initially provided ad libitum with a commercial glucose solution (BIOGLUC, Biobest) and pollen pellets (Biobest) accessible from within their nest box.

For conducting behavioural observations and to record flight and feeding sonication, we used flexible plastic tubing to connect the colony to a 100 cm × 60 cm × 35 cm flight arena made of wooden panels with a clear plastic top. In order to accustom the bees to foraging within the flight arena, we conditioned the bees using non-poricidal ‘training’ flowers. Prior to the start of the trials, three *Chrysanthemum* sp. (Asteraceae) flowers were presented within the arena and bees were allowed to forage freely. Training flowers were also present within the arena at all times when trials were not being carried out.

A day before each trial, pollen was not provided to the colony to encourage pollen foraging. For the trial, the training flowers were replaced by three *Solanum rostratum* (Solanaceae) flowers presented on a wooden stick. These flowers were grown in a pollinator-free environment at the University of Stirling glasshouses. For each trial, a single bee was allowed to enter the flight arena and forage for a maximum of 15 min or until it returned to the plastic tube and attempted to return to the colony. If a bee did not land on a flower after 10 min, the bee was returned to the colony and the next bee was allowed into the arena. Bees that visited flowers during their trials were collected, given a thoracic marking using water-based coloured pens (Posca, Mitsubishi, UK), and placed into a separate nest box within which they were kept for the remainder of the experiment. Flowers that had been visited were replaced with new, unvisited flowers after each trial. The marked individuals were then allowed on new foraging trials on the same or different days, and trials were continued until each bee had undergone ten trials.

We recorded flight and feeding sonication using a H4n Handy Recorder (Zoom, Weston, UK) placed 5 cm from the nearest flower (for detailed methods on acoustic recordings of this type see De Luca et al. 2014). The recorder was placed outside of the flight arena behind a fine plastic mesh. Sound was recorded throughout each 15 min trial, and for each trial, the date, the time of entry to the arena, the bee identity, arrival and departure times for each floral visit, and to which of the three flowers each visit was made were noted.

At the end of the experiment, bees were frozen and thorax width at the widest point was measured, as a proxy for bee size (Goulson et al. 2002). We analysed the sound data using the software package Audacity 2.0.5 (www.audacityteam.org). For each trial, we used a high pass filter with a roll-off of 12 dB per octave and a cut-off frequency of 100 Hz to reduce background noise. We then generated spectrograms from which the peak frequency and corresponding amplitude were identified for the first five clear (i.e., not obscured by background noise) sonication buzzes and the first five clear flight buzzes in each trial.

Data were analysed with general linear mixed-effects models implemented using the *nmle* package in *R* (Pinheiro et al. 2015; R Core Development Team 2015). Two sets of models were run in which the peak frequency of each buzz and the amplitude of each buzz were used respectively as response variables. Arena session, bee behaviour (flight or floral sonication) and a two-way interaction among both were included as explanatory variables, and bee identity was incorporated as a random effect in order to account for dependency among observations recorded from the same bee. Plotting the raw data indicated that there was no effect of either bee size (perhaps due to the limited range in variation in size in the individuals included in our trials; see Results) or flower position on frequency or amplitude of buzzes (data not shown) and these were therefore excluded from further analyses.

**Fig. 1:**
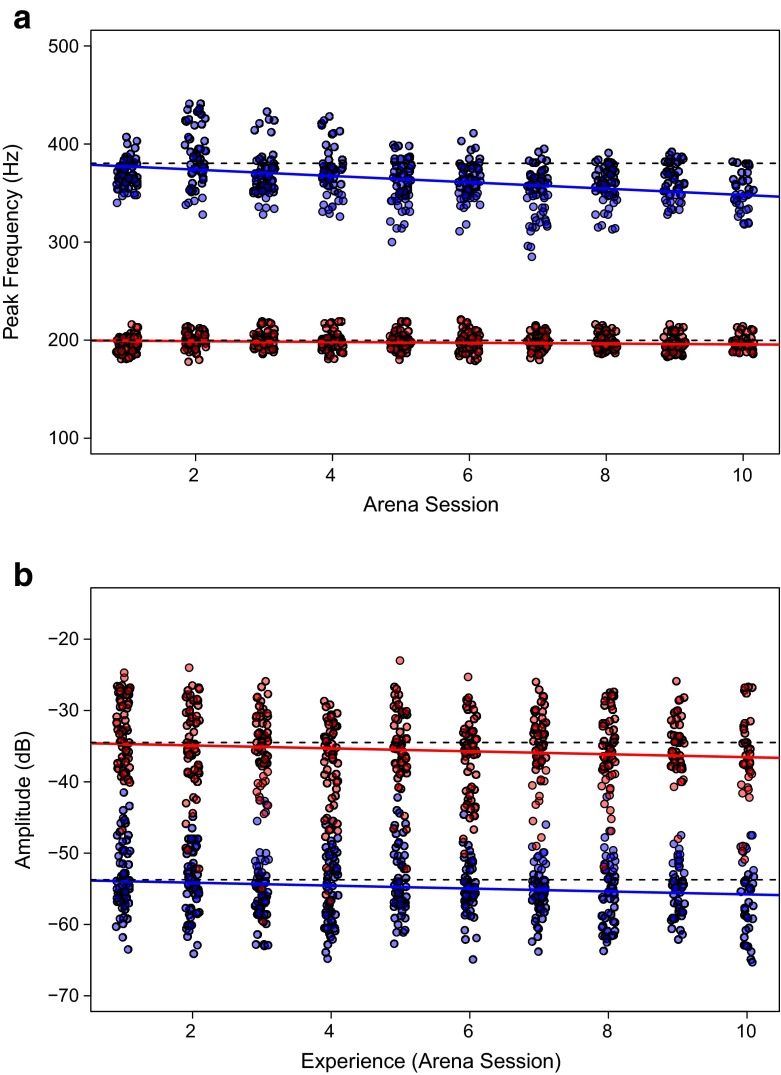
Relationship between **a** frequency and **b** amplitude of buzzes with accumulating experience. *Points* represent the raw data and *solid lines* represent regression lines for flight (*red*) and sonication (*blue*) buzzes obtained from the statistical analysis, and omitting non-significant interaction terms. *Broken lines* represent the intercept value for each buzz type. Horizontal jitter has been applied to facilitate interpretation of the figure

## Results

A total of 12 workers completed the 10 trials required to be included in the study. These bees varied in size with thorax widths ranging from 4.39 to 5.67 mm. When presented with *Solanum rostratum* for the first time, bees instinctively attempted to vibrate the flowers soon after entering the flight arena. Initially, bees tended to sonicate on the flower petals, but after two to three floral visits, they focused their sonication efforts exclusively on the anthers. The bees were able to remove pollen from the anthers, which was then packed onto their pollen baskets.

Flight and floral sonication buzzing were readily distinguishable from one another by both amplitude and frequency. Buzzing during flight was at a lower frequency (197.58 ± 8.8 Hz, mean ± s.d., *n* = 585 buzzes) than during floral sonication (362.7 ± 24.8 Hz, *n* = 584) (Fig. 1a, Table 1). Flight buzzes had higher amplitudes than floral sonication buzzes (−35.6 ± 5.82 dB and −54.86 ± 4.57 dB, respectively) (Fig. 1a, Table 1).

**Table 1:** Results of General Linear Mixed Effects Model (GLMM) analysis of peak buzz frequency (Hz) and amplitude (dB). Parameters were estimated using restricted maximum likelihood (REML). The *p*-values denote significant differences between floral sonication and flight buzzes; they were calculated using likelihood ratio tests based on maximum likelihood (ML) estimation. Slope represents the change in frequency or amplitude of the buzzes in consecutive trials in which bees gain experience at manipulating buzz-pollinated flowers. Numbers in brackets represent standard errors associated with the model parameters

	Intercept	*P*-value	Slope	*P*-value
Frequency				
Floral sonication	380.32 (2.59)	<0.0001	−3.23 (0.25)	<0.0001
Flight	199.79 (2.08)		−0.41 (0.35)	
Amplitude				
Floral sonication	−53.47 (0.66)	<0.0001	−0.26 (0.08)	0.3441
Flight	−34.76 (0.64)		−0.15 (0.11)	

The frequency of buzzes produced during floral sonication decreased as bees gained experience in handling the *S. rostratum* flowers (Fig. 1a, Table 1). The mean peak floral sonication frequency dropped from 369.0 ± 14.5 Hz (*n* = 60) during the first arena trial to 349.7 ± 19.1 Hz (*n* = 35) during the tenth. In contrast, the frequency of flight buzzes remained relatively constant over successive trials (195.9 ± 7.9 Hz versus 196.5 ± 8.6 Hz for trials 1 and 10, respectively) (Fig 1a, Table 1). Amplitude of both flight buzzing and floral sonication buzzes was reduced over the ten successive trials (−32.94 ± 4.97 dB to −36.55 ± 6.03 and −52.47 ± 5.83 dB to −56.33 dB ± 5.10 respectively, *p* < 0.001) (Fig. 1b). However, the effect of arena session on amplitude was the same for both flight buzzing and floral sonication buzzes (Table 1).

## Discussion

Although previous studies have demonstrated that bees can learn to recognise (Gegear and Laverty 2005; Raine et al. 2006) and manipulate flowers of different levels of complexity (Heinrich 1979; Laverty 1980, 1994), our study is the first to show that the characteristics of floral sonication change with increasing experience at manipulating flowers. By studying bumblebees from their first exposure to buzz-pollinated flowers, we observed that floral sonication is quickly attempted by naïve *Bombus terrestris* workers, thus corroborating the innate nature of this behaviour in at least some bumblebee species (Laverty 1980; King 1993). Despite this innate property, our analyses indicate that individuals of *B. terrestris* were able to modify the frequency of floral sonication buzzes as they learned to manipulate *Solanum rostratum.* In contrast, the vibrations produced during flight remained relatively consistent throughout the trials*.* Moreover, our study also verified that vibrations produced during flight and during floral sonication have clearly distinct acoustic signals, a feature that has been demonstrated in other bee species (King et al. 1996; Burkart et al. 2011). Altogether, our study indicates that learning may play a role in determining the specific characteristics of the sonication buzzes used by bees to extract pollen from buzz-pollinated plant species.

The characteristics of floral sonication buzzes, including number of buzzes, duration of each buzz, amplitude, and frequency, can influence the amount of pollen released from flowers (De Luca et al. 2013). Our study focused on two characteristics of floral sonication buzzes that have previously been shown to vary among individual bees and among bee species, namely frequency and amplitude (reviewed in De Luca and Vallejo-Marín 2013). Our results indicate that frequency changes as bees gain experience at manipulating buzz-pollinated flowers. The fact that the frequency of floral sonication drops with increasing experience is unexpected. It has previously been suggested that buzz-pollinated plants control the quantity of pollen removed per visit by being ‘tuned’ to higher frequencies than those of which bumblebees are capable (Harder and Barclay 1994). In that case, bees trying to extract more pollen from buzz-pollinated flowers should try to maximise the frequency of their floral sonication buzzes. However, De Luca et al. (2013) found little effect of frequency on pollen removal within the natural range of bumblebee sonication buzzes. The drop in frequency we observed in more experienced bees could be explained by a variety of mechanisms, which are not mutually-exclusive. First, it is possible that the reduction in the frequency of floral sonication buzzes represents ageing of the bees over the duration of the experiment rather than a modification of buzzes due to learning. The average number of days to complete the ten trials for each bee was 9.41 days (range: 6–15 days). We think the ageing explanation is unlikely because we observed a negligible reduction in the frequency of flight buzzes of the same bees measured in the same conditions. In this regard, the measurement of flight buzz frequencies served as an internal control for changes in sonication characteristics unrelated to floral manipulation. Second, the drop in frequency may represent a mechanism to optimise energy expenditure during pollen extraction. Given that variation in frequency within the sonication range of *B. terrestris* has little effect on the amount of pollen released by *S. rostratum* (De Luca et al. 2013), then, all else being constant, a reduction in frequency, would allow bees to spend less energy collecting a similar amount of resource. Future experiments examining the amount of pollen collected per flower by sonicating bees of increasing experience are needed to test this hypothesis.

Amplitude has a significant effect on pollen removal in interactions between *S. rostratum* flowers and vibrations in the range of *B. terrestris*, with higher amplitudes resulting in substantially greater pollen release (De Luca et al. 2013). Given this, we expected that amplitude of floral sonication buzzes should increase as bees gain increasing experience removing pollen from *S. rostratum* flowers (alternatively amplitude should stay the same if bees are already producing sonications with maximum amplitude from the start). However, contrary to this expectation, we found that amplitude of floral sonication buzzes decreased over successive arena sessions. This reduction in amplitude may be unrelated to pollen collection as suggested by the fact that amplitude is also reduced in flight buzzes. Such reduction of amplitude across behaviours may simply reflect ageing of the bees. An intriguing possibility is that, if a reduction in amplitude is accompanied by lower sonication frequencies, bees may in fact be able to optimise pollen removal. King and Buchmann (1996) found that the amplitude required for releasing pollen from poricidal anthers increases with increasing frequency. Therefore, counterintuitively, reducing the frequency of floral sonication buzzes could trigger pollen release at lower amplitudes.

Further work will be required to establish how the changes in floral sonication properties observed in this study affect pollen release and collection. Future studies would also benefit from considering how morphological characteristics of the flower, such as anther mass and the size of the anther pores, affect the relationship between sonication and pollen release. Although, the generality of our findings remains to be assessed in other bee species and in other buzz-pollination systems, our study presents an intriguing insight into a previously unexplored aspect of bumblebee learning and the interactions between buzz-pollinated plants and their pollinators.
